# Clinical trial to test the safety of the EVA Nexus surgical platform

**DOI:** 10.1186/s40942-024-00563-3

**Published:** 2024-06-24

**Authors:** Stalmans Peter

**Affiliations:** Department Ophthalmology UZLeuven, Herestraat 49, Leuven, 3000 Belgium

**Keywords:** Vitrectomy device, Vitrectomy safety, Latrogenic complications, EVA NEXUS surgical system

## Abstract

**Background:**

The EVA Nexus system offers several technical improvements over its predecessor. The newly designed Aveta cannula system for vitrectomy surgery avoids the need for removal of the valve from the infusion cannula. The chamfered leading edge of the cannula also reduces the insertion force needed. The new EquiPhaco needles in combination with SmartIOP provide excellent anterior chamber stability during phaco-emulsification surgery, enabling to work at lower infusion pressures, and the multiburst phaco mode allows easier removal of hard cataracts. The system offers a secondary active infusion line for independent control of pressure to the anterior and posterior chambers, monitoring of flow rate/reflux and warning of infusion bottle emptying. This study evaluated whether these technical improvements result in improved surgical safety.

**Methods:**

In total, 250 eyes that underwent vitrectomy (53%) or phaco-vitrectomy (47%) using the EVA Nexus system were prospectively included. The occurrence of intraoperative adverse events was compared to that of historically operated eyes using the EVA system.

**Results:**

The average age of the patients was 63 years. A total of 33% of the patients were operated on for retinal detachment, 17% for macular pucker, 11% for treating floaters, 9% for removing silicone oil, 8% for macular hole repair and 22% for other diseases. In 75% of surgeries, 23 G instruments were used, and 27 G instruments were used in 25% of cases. Device issues that occurred included priming cycle issues (*n* = 4), eye pressure stability problems (*n* = 6) and vitrectome performance issues (*n* = 1), all of which in the first 100 patients who were included and were fixed with software updates. The frequency of surgical complications in the anterior segment was lower than that in the historically recorded surgical reports. Intraoperative events in the posterior segment included hemorrhage from retinal vessels, choroidal hematoma, iatrogenic retinal damage/tear, and subchoroidal infusion. Again, these events occurred rarely and less frequently than in the historical surgical reports.

**Conclusions:**

The EVA Nexus provides a surgical platform that reduces the incidence of intraoperative adverse events and iatrogenic complications in both anterior and posterior segment surgery. This could increase surgical safety during cataract and vitrectomy surgery.

**Trial Registration number Clinicaltrials.gov:**

: NCT05229094 Data 22/5/2021.

## Background

To perform phaco-emulsification and vitrectomy surgery, a device is required to provide the required amount of ultrasound power, infusion flow and aspiration rate during all steps of the surgery. Several devices are available on the market that offer the combined functionality of phaco-emulsfication and vitrectomy surgery. All these devices have different modules for this purpose [1]:

- An infusion system to pressurize the anterior chamber or vitreous cavity during the surgery. This infusion system can be passive using gravity whereby the infusion bottle is elevated to increase the intra-ocular pressure, can be active by pumping air into the infusion bottle that generates the required infusion pressure, or can be pump-driven whereby a pump aspirates fluid from the infusion bottle (or bag) and pushed the fluid in the eye.
- An aspiration system that extracts lens material / vitreous / liquids / silicone oil from the eye. Aspiration can be generated by different pump types. In a venturi system, a vacuum is created that generates secondary flow in the aspiration line. In a peristaltic system, a rotary pump moves fluid through a tubing that can generate vacuum in case the tip of the aspiration line is (partially) occluded. Finally, a piston pump with two valves and chambers can either behave as a vacuum system (such as a venturi pump) or can be software-steered to behave as a flow system (such as a peristaltic system).
- A vitrectome, which is an air-pressure driven device that removes vitreous from the eye with a small guillotine knife at its tip. The surgeon can control the speed of the back-and-forth movement of the knife (cut speed). In older vitrectome designs, the tip was occluded with every back-and-forth movement of the knife. In more modern vitrectomes, the knife has an opening which not only prevents occlusion of the tip when in motion, but also generates two cutting actions in each cycle. This way, cut speeds of up to 20 000 cuts-per-minute are nowadays available.
- A phaco module which generates ultrasound power on a metal tip surrounded by and infusion sleeve which allow emulsification of the lens. Different needle diameters, angulations and tip bevels are available. By using pulsed delivery of the ultrasound power, not only can the amount of total energy in the eye be reduced, but this also avoids repulsion of the lens fragments during the phaco-procedure.
- A light source on which a light fiber is connected to illuminate the inside of the vitreous cavity. Several years ago, halogen light was used for this purpose. Later on, more powerful Xenon light became available, which is now being replaced by LED light sources which have a much longer life span.
- A laser module to perform retinal laser treatment. Without exception, all available devices on the market have a 532 nm laser built-in.
- A module to generate pressure or vacuum to inject or aspirate silicone oil in/from the eye.

Table 1 show an overview of the different devices available on the (European) market.

**Table 1:** Overview of vitrectomy devices on the market

Brand	Name	Aspiration system	Infusion system	Illumination
Alcon	Constellation	Venturi	Air-driven	Xenon
Bausch + Lomb	Stellaris PC	Venturi	Air-driven	Xenon
BVI	R-Evolution CR	Peristaltic + Venturi	Gravity or pump-driven	LED
DORC	EVA	Piston pump	Air-driven	LED
DORC	EVA NEXUS	Piston pump	Pump-driven	LED
Oertli	Faros	Peristaltic – SPEEP	Gravity or air-driven	LED

The EVA Nexus (DORC, Zuidland, The Netherlands) is a novel surgical platform that became available in 2021 for anterior and posterior segment eye surgery. Compared to its predecessor (DORC EVA), many improvements were made:

- The advent of 3D surgery requires a different layout for the whole surgical room. Therefore, device ergonomics were improved by fitting a screen that can be rotated and angled in any desired position to optimize viewing by the surgeon regardless of his or her position relative to the device. Additionally, a brake level both in front of and behind the device allows control by both the scrub nurse and handling nurse.
- The new Aveta cannula set was improved in several ways: the outer diameter of the entry funnel was reduced in size to increase the range of eye maneuvering during the surgery. The shaft of the cannula is laser-etched to improve its retention in the eye. Finally, the infusion line can be connected without the need to remove the valve cap from the cannula. This not only reduces the amount of manipulations during the surgery but also allows quick connection of the infusion line on a different cannula when needed. Although the Aveta was introduced at the same moment of the availability of the Nexus system, it is also available as a separate set to use with the previous EVA system.
- A new range of phaco needles is available, with 4 diameters (1.8, 2.2, 2.4 and 2.8 mm), two curvatures (angled or straight) and two tip types (with a 30 or 45 degree bevel), for a selection of 16 different needles. For all tips, the sleeve diameter is the same as the maximal tip size, indicating that the sleeve can smoothly enter the anterior chamber through the incision without curling. The wide selection of needles accommodates the preference of each individual surgeon.
- The aspiration pump still offers dual control (flow control or vacuum control) similar to the DORC EVA VTi pump but now includes flow limitation in vacuum mode for greater control of the flow rate postocclusion. Together with improved pump steering, very precise flow control can be obtained, even when air is being aspirated.
- Whereas the EVA device pressurizes the infusion bottle to generate the required amount of intraocular pressure (IOP) requiring rigid glass bottles, the EVA Nexus has a secondary pump for this purpose. This has several advantages. First, the system can work with balanced salt solution (BSS) in glass or plastic bottles and bags. Second, the distance to the area in the system where the pressure is generated is shortened, which results in more precise and faster control of the IOP. Finally, the dual pump system (one for aspiration and one for irrigation) enabled the development of an algorithm that predicts the amount of irrigation required to compensate for the actual infusion. This algorithm, named “SmartIOP”, provides improved IOP stability during surgery and can be used to control irrigation/infusion independently in both anterior and posterior segment surgery.
- The amount of fluid left in the infusion bottle is displayed on the device screen. When the bottle is nearly empty, a warning is shown to prevent the system from running without irrigation fluid.
- The actual infusion flow can be monitored on the screen, displayed in ml/minute.

This study examines the surgical safety by comparing the occurrence of surgical adverse events between the EVA and the EVA Nexus platform.

## Methods

A prospective, investigator-initiated, academic, monocenter field observation study using the EVA Nexus surgical device was designed to evaluate the safety of this new surgical platform. In a prospective study arm, a total of 250 patients were enrolled. The study procedure consisted of three patient visits: a preoperative visit, the surgery itself and the postoperative follow-up visit the day after the surgery. As standard-of-care during surgery, intraoperative complications and device issues were recorded for safety assessment. To record the surgical complications, the classification system published previously by Tim Jackson et al. was used [2–6] (Table 2). Typical postoperative follow-up after vitrectomy included clinical tests performed on the first postoperative day to assess whether a normal postoperative outcome was present without the presence of adverse events.

**Table 2:** List of adverse events recorded during (phaco)vitrectomy

Anterior segment surgery	Posterior segment surgery
Anterior capsule zip	Latrogenic retinal tears
Posterior capsule tears	Lens touching
Vitreous prolapse	Choroidal hematoma
Iris prolapse	Subchoroidal infusion
Dropped nucleus	Latrogenic retinal damage
	Subretinal hemorrhage
	Iris trauma
	Retinal incarceration
	Subretinal perfluorocarbon liquid
	Subretinal silicone oi
	Hemorrhage from retinal vessels

In a retrospective comparative study arm, the same clinical data were collected from a historical cohort of patients operated with the EVA platform, both using 23G and 27G instruments. This allowed us to compare the incidence of surgical complications and device issues to surgeries performed earlier using the DORC EVA platform). To collect these historical data, a query was performed in the electronical medical records of the hospital to retrieve the vitrectomy surgical reports between Oct 2020 and July 2022 from the same surgeon (P.S.) performed with the DORC EVA platform since the time when surgical complications and device issues were recorded in the reports.

All surgeries were performed by one surgeon (P.S.). As a standard-of-care, macular surgeries (for macular hole, macular pucker and vitreomacular traction) are performed with 27G cannulas and instruments, while all other surgeries (e.g. retinal detachments, diabetic retinopathy, oil removal) are performed with 23G cannulas and instruments. The reason to use 23G instruments for the more complicated cases is that injection and extraction of (heavy) liquids is easier through a larger lumen, avoiding a high jet-flow that can penetrate the retina. Moreover, not all instruments required for membrane peeling (e.g. membrane spatula, curved scissors) are available in 27G. This gauge choice was not different between the EVA and EVA Nexus platform. The funnel design of the new Nexus – Aveta cannulas is different (smaller) compared to the design of the older EVA cannulas, but both outer diameter and length remained the same and both are valved cannula systems.

In case of combined phaco-vitrectomy surgery with the DORC EVA platform, a curved phaco needle of 1.8 mm was used with a 30 degree beveled tip. When surgery was performed using the DORC EVA Nexus, a straight phaco needle of 1.8 mm with a 45 degree bevel (EquiPhaco type) was used in all cases.

Patients aged 18 years who were scheduled for vitrectomy surgery or combined surgery, regardless of the indications, were included. Eyes undergoing primary or repeat vitrectomy were operated on using the EVA Nexus system. Surgery was performed under general or local anesthesia, the latter of which could also be administered under sedation.

At the time of the study, the EVA Nexus device had already received a CE-Mark; hence, no permission from the Competent Authorities in Belgium was required to use the device for this study.

Approval from the UZLeuven Ethics Committee to conduct this study was received on April 1, 2021 (study reference number S64913), prior to patient inclusion. All prospectively included patients signed an informed consent and approval form. All procedures performed were in accordance with the ethical standards of the institutional and/or national research committee and with the 1964 Helsinki declaration and its later amendments or comparable ethical standards.

The study data described in Table 3 were pseudonymized and entered into the RedCap eCRF database (Vanderbilt University), then anonymized and exported for statistical analysis (Ars Statistica, Nijvel, Belgium).

**Table 3:** Propensity score

	Before matching			After matching		
	DORC EVA (*n* = 457)	DORC EVA Nexus (*n* = 250)	ASD	DORC EVA (*n* = 457)	DORC EVA Nexus (*n* = 250)	ASD
Age	61.56 (15.5)	63.14 (12.1)	11.34%	62.22 (14.85)	62.22 (12.79)	0.01%
Vitrectomy	65.86%	53.20%	26.02%	61.02%	61.01%	0.01%
Macular Surgery	16.19%	26.40%	25.13%	19.94%	19.94%	0.00%
Retinal Detachment Surgery	43.33%	34.40%	18.39%	39.85%	39.85%	0.00%
Oil Removal	8.10%	9.20%	3.93%	8.39%	8.39%	0.00%
Floaters	7.88%	11.20%	11.33%	9.20%	9.20%	0.00%
Other	24.51%	18.80%	13.89%	22.63%	22.63%	0.00%
No tamponade	38.95%	44.00%	10.26%	40.62%	40.62%	0.00%
Air	6.35%	8.40%	7.87%	7.27%	7.27%	0.00%
Gas	33.04%	32.80%	0.51%	33.26%	33.27%	0.00%
Silicone oil	21.66%	14.80%	17.85%	18.85%	18.85%	0.01%
Eye (OD)	52.08%	49.60%	4.96%	50.11%	50.10%	0.01%

ASD = Absolute Standardized Difference

### Statistical methods [7–15]

For the primary endpoint (complication rate of the EVA Nexus), a one-sample proportion test was performed to test whether the obtained complication rate was different from rates found in the literature (30%, 15% and 5%). For the remaining data (demographics and secondary objectives), group comparisons (no complications vs. complications) of categorical count-data variables were performed using Fisher’s exact test, and group comparisons (no complications vs. complications) of continuous data variables were performed using the T test or the Wilcoxon signed-rank test, as appropriate (T test when the normality of the residuals and the equality of the variances are met, and the Wilcoxon signed-rank test when the normality of the residuals or the homoskedasticity of the variances are not met). Finally, to test the first predictive model, a logistic or Poisson regression, as appropriate, with the backward selection method was performed, starting with all variables associated with the target with a p value lower than 0.1. The final model was constructed from variables associated with the outcome with a p value lower than 0.05. An overview is presented in Table 3.

## Results

### Demographic data

In total, 250 patients were included in the prospective study arm and operated with the EVA Nexus system between May 2021 and April 2022; the male/female ratio was 53/47%, and the OD/OS ratio was 124/126 eyes. The median age was 63 years (range 19–95). In 127 eyes (51%), previous ocular surgery had been performed, mostly cataract surgery (109 eyes, 44%). The most frequent indication for vitrectomy was retinal detachment (33%), followed by macular pucker (17%), floater treatment (11%), silicone oil removal (9%) and macular hole repair (8%). Most surgeries were performed under general anesthesia (86%), while retrobulbar anesthesia was used in 12%, administered after sedation in 2%.

In 53% of eyes, only vitrectomy surgery was performed, as the patients were either pseudophakic or younger than 50 years. In 47% of eyes, combined phaco-vitrectomy was performed, for whom a monofocal spherical IOL (CT Asphina 409, Zeiss, Oberkochen, Germany) was typically used. In cases of astigmatism of > 1.75 diopters, a monofocal torical IOL was chosen (AT Torbi, Zeiss). Among eyes where good recovery of vision was expected (e.g., macula-on or recently macula-off detachments, floater removal surgery), the use of an EDOF IOL (AT LARA, Zeiss) was suggested and implanted in 10% of cases, and the toric version (AT LARA toric, Zeiss) was used when the astigmatism exceeded 1 diopter (5% of eyes). In cases requiring a (toric) EDOF IOL, a capsule tension ring was always implanted (ACPI 11, Bausch + Lomb, Vaughan, Ontario, Canada).

Most surgeries were performed using 23 G instruments (75%), while the rest were performed with 27 G instruments, mostly macular surgeries or floater removals [15]. Vital dyes were used in 147 surgeries, including ILM Blue (DORC) in surgeries for macular holes or vitreomacular traction, Membrane Blue (DORC) in surgeries for epiretinal membranes or the presence of proliferative vitreoretinopathy membranes, and triamcinolone (Triesence, Alcon, Rochester) for staining the vitreous during retinal detachment surgery [16]. Suturing was required to close the sclerotomies in 21% of eyes [17].

In the retrospective historical study arm, 457 patients were included who underwent surgery with the EVA system. To determine the Propensity score, the data of both study arms were matched to indication of surgery (Table 4). The standardized difference values indicate that the two groups have equal means/proportions for the different variables after matching. Both study arms (DORC EVA and DORC EVA Nexus) can be considered similar on covariates chosen for the propensity score.

**Table 4:** Incidence of surgical and device adverse events. Significant p values are indicated with an asterisk

Variable	DORC EVA (*n* = 457)	DORC EVA Nexus (*n* = 250)	*p* value
**Surgical complications:**			
*Anterior segment*:			
Lens capsule zip	0.56%	0.37%	0.7204
Lens capsule tears	3.14%	2.08%	0.3742
Iris prolapse	0.65%	0%	0.0825
Vitreous prolapse	0.47%	0%	0.154
*Posterior segment*:			
Hemorrhage from retinal vessels	2.94%	0.74%	0.02226
Choroidal hematoma	0.66%	0.87%	0.7775
Dropped nucleus	1.02%	0.28%	0.1991
Iatrogenic retinal damage	0.55%	0%	0.155
Iatrogenic retinal tears	1.28%	0%	0.0145*
Infusion subchoroidal	1.48%	0.62%	0.2969
Lens touching	0.56%	0%	0.0829
**Device issues:**			
Cannula problems	4.37%	2.74%	0.2552
Empty infusion bottle	1.81%	0%	0.026*
Illumination problems	0%	0.95%	0.167
Irrigation problems	6.54%	3.26%	0.0400*
Air bubbles in the eye	4.54%	0.95%	0.0015*
Vitrectome problems	0.22%	0.69%	0.377

### New device features recorded by the surgeon

As mentioned above, the EVA Nexus platform offers the possibility to see the actual infusion flow during the procedure. During the course of the study, it was found that this surgeon feedback system offers two added safety features. First, at the onset of core vitrectomy, a quick glance at the infusion flow provides confirmation for the surgeon that the infusion cannula is not blocked by a vitreous wick. Second, when injecting perfluoro-carbon liquid (PFCL) in the eye during retinal detachment surgery, a negative infusion flow on the display confirms successful outflow of infusion liquid through the infusion line, eliminating the risk of increased IOP during this surgical maneuver.

### Device issues during surgery

Device issues were recorded as follows: in four surgeries, a device deficiency occurred; in three surgeries, repriming was required to initiate the surgery; in one surgery, a complete reboot of the system with new tubing was required when a fatal error message appeared during the surgery; in two surgeries, the eye pressure was too low and needed to be increased to stabilize the IOP; and in four surgeries, the infusion line stopped functioning, requiring reactivation. None of these device issues led to adverse events in the patient. All of them occurred in the first 100 patients included and were resolved by software updates during the course of the study. Other device-related issues recorded included air bubbles that entered the eye through the infusion line in six cases, a lost cannula in three cases, a light fiber that was not recognized in 2 cases and a faulty vitrectome during 1 surgery.

### Intraoperative complications

Adverse surgical events, as listed in Table 4, were also recorded for all surgeries. Since these events have been recorded in our surgical reports for many years, a comparison was made to 457 eyes that were operated on using the DORC EVA system between October 2020 and December 2021 (Table 4). These surgical adverse events were rare and tended to occur less frequently in eyes operated on using the EVA Nexus system. Two complications, namely, iatrogenic retinal tear and subchoroidal hemorrhage, even occurred at significantly reduced incidence.

## Discussion

The (phaco)vitrectomy surgeries in this study were performed using the new EVA Nexus, which had several technical improvements to increase surgical safety. A new feature of the EVA Nexus platform that allows monitoring of the infusion flow was found by the surgeon to be a valuable tool during the surgery. In this clinical study, only a small number of device issues occurred, which were resolved by software upgrades during the course of the study and did not reappear during the surgeries performed on the second half of the prospectively included patients. Surgical adverse events in general occurred rarely during (phaco)vitrectomy but seemed to have a lower incidence when performed using the EVA Nexus device than with its predecessor.

The SmartIOP feature of the EVA Nexus provides an automatic stabilization of the anterior chamber during phaco surgery, reducing the occurrence of collapse of the anterior chamber during the procedure. In a teaching hospital such as the UZLeuven, it was found that this feature was very beneficial when training residents in phaco surgery, since the SmartIOP reduces the risk of perforating the posterior lens capsule with the phaco tip.

As shown in Table 4, some surgical and technical issues occurred significantly less in the EVA Nexus system compared to the DORC EVA system. Firstly, there were less iatrogenic breaks. Although the vitrectome aspiration system (VTi pump) remained unaltered, this difference may be related to more precise steering of the infusion liquid providing a more stable eye pressure leading to more stability of the retina in surgery for retinal detachment during vitreous shaving. Secondly, several technical infusion problems occurred less frequently such as undetected empty bottle, irrigation problems in general (e.g. blocked infusion due to vitreous clogging) and air bubbles being injected in the eye through the infusion line. This is clearly a direct result of the newly designed active infusion system that also allows (automatic) monitoring of the bottle volume and of the infusion flow by the surgeon.

A limitation of this study is that only the data from the EVA Nexus system were collected prospectively, while the surgical data from the previous EVA system were retrospectively collected. Also, there were no data collected to compare the EVA Nexus platform to other surgical platforms on the market. Furthermore, data collection was only performed till the first postoperative day. Hence, long-term complications such as infection or retinal (re)detachment were not recorded.

Although the incidence of adverse events recorded with the predecessor EVA surgical system was already very low, this study highlights that the multiple improvements introduced into the EVA Nexus all contributed to marginal reductions in the frequency of complications. Since the major improvement of the EVA Nexus system is the very precisely controlled active infusion system, it is most probable that this feature contributes mostly to the increased safety of surgery. However, independently, these improvements may seem unsubstantial; collectively, they represent important progress in clinical goal of reducing the risks of iatrogenic complications.

## Conclusions

This prospective clinical study indicates that the EVA Nexus surgical platform has an excellent safety profile for surgeries performed in both the anterior and posterior eye segments.

## Data Availability

The source dataset(s) supporting the conclusions of this article are available in the patient electronical medical record repository of the UZLeuven (KWS ‘Clinical Workstation’).
